# Circulating serotonin and dopamine concentrations in osteoarthritis patients: a pilot study on the effect of pelotherapy

**DOI:** 10.1007/s00484-023-02571-8

**Published:** 2023-11-14

**Authors:** Isabel Gálvez, María Dolores Hinchado, Eduardo Otero, María Carmen Navarro, Eduardo Ortega-Collazos, Leticia Martín-Cordero, Silvia Teresa Torres-Piles, Eduardo Ortega

**Affiliations:** 1 Immunophysiology Research Group, Instituto Universitario de Investigación Biosanitaria de Extremadura (INUBE), 06006 Badajoz, Spain; 2 Departamento de Enfermería, Facultad de Medicina y Ciencias de la Salud, Universidad de Extremadura, 06006 Badajoz, Spain; 3 Departamento de Fisiología, Facultad de Ciencias, Universidad de Extremadura, 06071 Badajoz, Spain; 4 Servicio de Medicina Interna, Hospital San Pedro de Alcántara, 10003 Cáceres, Spain; 5 Departamento de Enfermería, Centro Universitario de Plasencia, Universidad de Extremadura, 10600 Plasencia, Spain; 6 Departamento de Terapéutica Médico-Quirúrgica, Facultad de Medicina y Ciencias de la Salud, Universidad de Extremadura, 06006 Badajoz, Spain

**Keywords:** 5-HT, Mud therapy, Pelotherapy, Hydrotherapy, Spa therapy, Inflammation

## Abstract

Balneotherapy has demonstrated clinical efficacy in the management of pathologies involving low-grade inflammation and stress. In rheumatic conditions such as osteoarthritis (OA), this therapy presents anti-inflammatory properties and potential to improve psychological well-being. Although the neurohormones serotonin and dopamine are known to be involved in these processes, surprisingly they have not been studied in this context. The objective was to evaluate the effect of a cycle of balneotherapy with peloids (pelotherapy) on circulating serotonin and dopamine concentrations in a group of aged individuals with OA, after comparing their basal state to that of an age-matched control group. In our pilot study, a pelotherapy program (10 days) was carried out in a group of 16 elderly patients with OA, evaluating its effects on circulating serotonin and dopamine concentrations (measured by ELISA). Individuals with OA showed higher levels of serotonin and lower dopamine levels, in line with the inflammatory roles of these mediators. After pelotherapy, serotonin concentrations significantly decreased, potentially contributing to the previously reported anti-inflammatory effects of balneotherapy.

## Introduction

Balneotherapy is a set of methods in which mineral waters, muds, and natural gases from natural springs (with medical and legal recognition) are used for therapeutic applications. Particularly, muds, also known as peloids, are maturated muddy suspensions consisting of a complex mixture of fine-grained geologic materials, mineral water, and, often, organic and biological compounds. Thus, mud therapy or pelotherapy is a balneological intervention in which mud is applied externally for therapeutic purposes (Gomes et al. 2013). These therapies can be applied in the context of spa therapy programs at thermal spa centres (Cozzi et al. 2018). Owing to its anti-inflammatory, antioxidant, and chondroprotective properties, balneotherapy has shown clinical effectiveness as a complementary treatment for various pathologies involving low-grade inflammation and stress, with a special emphasis on rheumatic conditions (Gálvez et al. 2018a; Cheleschi et al. 2022a), such as ankylosing spondylitis, rheumatoid arthritis (RA), fibromyalgia, and osteoarthritis (OA) (Cozzi et al. 2018; Fioravanti et al. 2018; Gálvez et al. 2018a).

OA is a degenerative disease that represents one of the major causes of disability and is prevalent among aged patients with multiple comorbidities and increased risk of adverse outcomes linked to polypharmacy (Antonelli et al. 2018; Cozzi et al. 2018; Hunter and Bierma-Zeinstra 2019). First-line treatments are non-pharmacological methods such as self-management and education, exercise, walking aids, and weight loss when necessary. However, pain medication is frequently required (Hunter and Bierma-Zeinstra 2019). In the recent past years, numerous studies assessing balneotherapy and pelotherapy in the treatment of OA have demonstrated their safety, showing minimal to no adverse effects, as well as their efficacy, resulting in pain and stiffness reduction, decreased drug consumption, and improved quality of life (D'Angelo et al. 2021; Ortega et al. 2017; Antonelli et al. 2018; Cozzi et al. 2018). In OA patients, pelotherapy exerts its beneficial effects through the stabilization of neuroendocrine-immune interactions. This process involves circulating inflammatory cytokines, cortisol, and extracellular heat shock proteins (eHsp72), together with changes in regulatory T cell phenotype and modulation of innate responses mediated by neutrophils and monocytes (Ortega et al. 2017; Gálvez et al. 2018b, 2020). Despite these recent advances, there is a notable lack of research examining the effects of balneotherapy on other physiologically relevant neurohormones, such as serotonin and dopamine, involved both in psychological and neuroimmunological regulation with potential capacity for modulating the inflammatory response, as reviewed by our research group. By elucidating these mechanisms, we may uncover the broader applications of these therapies in the management of various pathologies associated with serotonin and/or dopamine dysregulation.

Serotonin (5-hydroxytryptamine, 5-HT) is a neurotransmitter that mediates a wide range of biological and behavioural processes including pain, cognition, mood, and anxiety/stress. Outside the central nervous system, peripheral serotonin acts as a hormone and plays a key role in many other physiological functions including tissue regeneration, and immunity and inflammatory modulation (Wu et al. 2019; Nieto et al. 2021). In physiological conditions, the systemic levels of serotonin are low. However, during inflammatory processes, serotonin is released from platelets, where they are mainly stored, leading to a significant increase in its plasma concentration, especially around sites of inflammation (Casas-Engel and Corbí 2014). In this context, altered peripheral serotonin levels have been demonstrated to be involved in the development and resolution of immunity and inflammation-related pathologies, including arthritis (Nieto et al. 2020; 2021). Consequently, researchers and clinicians have suggested peripheral serotonin as a novel target for the treatment of different immune/inflammatory diseases (Banskota and Khan 2022). However, the role of peripheral serotonin in OA and its potential physiological variations in response to non-invasive and non-pharmacological anti-inflammatory strategies such as balneotherapy and pelotherapy has not yet been studied.

Dopamine, or 3-hydroxytyramine, is a catecholamine neurotransmitter with crucial functions in a range of processes in the central nervous system, such as motor control, cognition, emotion, and behaviour (Matt and Gaskill 2020; Feng and Lu 2021). Apart from this, dopamine is also present in most of peripheral tissues, regulating numerous physiological functions including immune function (Pinoli et al. 2017; Matt and Gaskill 2020). In fact, recent research has emphasized the relevance of dopaminergic pathways in OA pathophysiology (Sheikhpour et al. 2020). Furthermore, dopamine has emerged as a potential novel therapeutic agent in OA (Lu et al. 2019). Therefore, it is plausible to consider the possibility of dopamine being involved in the inflammation-mediated benefits of pelotherapy in inflammatory pathologies such as OA.

Taking all of this into account, and complementing a previous review on the role of spa therapy and peripheral serotonin and dopamine function, the objective of this pilot study was to assess the effect of a cycle of balneotherapy with peloids (pelotherapy) on circulating serotonin and dopamine concentrations in a group of elderly patients with OA, particularly whether this intervention could improve potential altered systemic concentrations of these neurohormones in these patients. Then, prior to this, it was necessary to assess the basal status of these patients in relation to an age-matched control group. To the best or our knowledge, this is the first experimental approach evaluating the effect of pelotherapy on changes in circulating concentrations of serotonin and dopamine.

## Material and methods

### Experimental design and participants

A prospective, interventional study was conducted in a group of OA patients. Both experimenters and researchers were blinded since both the experimental and control groups were assigned computer-generated random alphanumeric codes. The study was carried out at the spa centre “El Raposo” (Puebla de Sancho Pérez, Badajoz, Spain), during a 10-day cycle of balneotherapy with peloids, following the same experimental design as previous studies from our group (Ortega et al. 2017).

Inclusion criteria required participants to be 60 years old or more and diagnosed with primary knee OA by a rheumatologist based on the ACR criteria (Altman et al. 1986).

Exclusion criteria were presence of musculoskeletal, neoplastic, cardiological, vascular, respiratory, or immune (including inflammatory) conditions; knee replacement patient; consumption of non-steroidal anti-inflammatory drugs (NSAIDs) within the last 3 days; intra-articular injections of corticosteroids or hyaluronic acid within the past 6 months; and therapy with corticosteroid or anticytokine drugs, either oral or local.

**Fig. 1:**
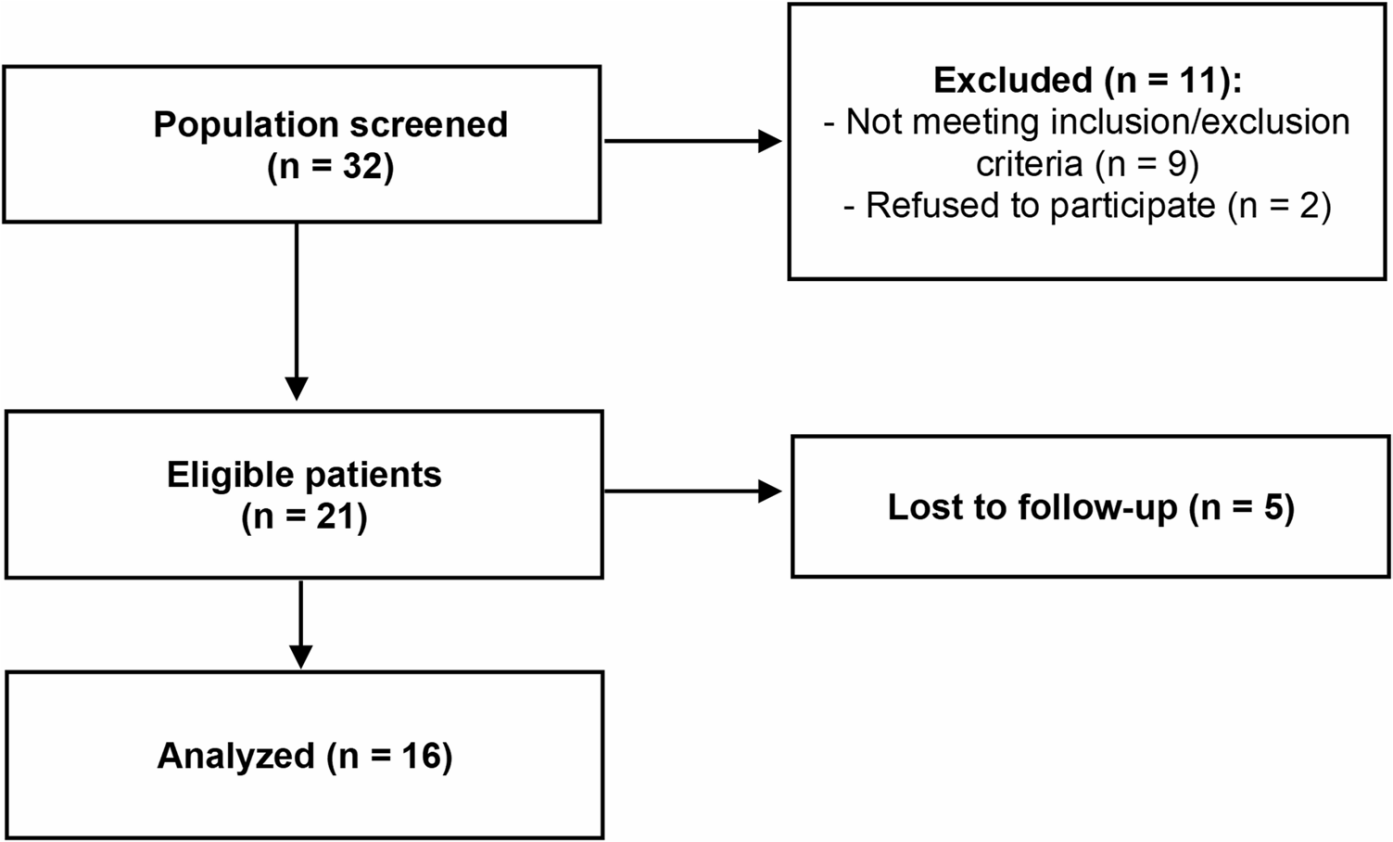
Flow of participants throughout the study

Thirty-two participants were screened, and after the exclusion of eleven participants (nine did not comply with inclusion/exclusion criteria and two refused to participate), finally 21 patients were eligible for enrolment in the investigation. Five patients withdrew during the study (they left the spa centre at the end of their stay, before post-treatment evaluation, due to personal reasons), leaving a final sample size of 16 patients. Sixteen volunteers were enrolled in the study after being informed of the investigation (Fig. 1). Their ages ranged from 60 to 77 years, with a mean age of 69.25 ± 1.47 years. All participants provided written informed consent before taking part in the study. Alphanumeric codes were used to identify each volunteer to preserve their anonymity. Approval for the study was granted by the Ethical Committee of the University of Extremadura, Spain (036–15), following the principles and regulations of the European Community Council Directives and the Helsinki Declaration.

Following the same exclusion criteria, eight healthy individuals constituted the age-matched control group, with an age range of 63 to 71 years old and a mean age of 67 ± 1.08 years. Control subjects belonged to the same region and shared a similar lifestyle as the OA group. Demographic and anthropometric data of all participants are presented in Table 1.

**Table 1:** Demographic and anthropometric data

	Age (years)	Weight (kg)	BMI (kg/m^2^)	WHR
Total OA patients (*n* = 16)	69.25 (± 1.47)	76.78 (± 2.5)	30.02 (± 0.94)	0.88 (± 0.02)
• OA men (*n* = 8)	68.37 (± 2.09)	80.67 (± 2.9)	30.83 (± 1.16)	0.93 (± 0.02)
• OA women (*n* = 8)	70.12 (± 2.18)	72.9 (± 3.82)	29.19 (± 1.54)	0.81 (± 0.01)
Total control (*n* = 8)	67 (± 1.08)	69 (± 4.76)	26.62 (± 1)	0.89 (± 0.02)
• Control men (*n* = 2)	66 (± 1)	86 (± 7)	29.44 (± 2.73)	0.99 (± 0.03)
• Control women (*n* = 6)	67.33 (± 1.42)	63.33 (± 3.65)	25.69 (± 0.81)	0.85 (± 0.02)

Data are expressed as mean (± SEM). *BMI* body mass index, *WHR* waist-hip ratio

Blood samples from the OA group were obtained in basal state, that is, 1 day after arrival to the spa centre, just before the first session of balneotherapy (pre-treatment). Pre-treatment values were compared to those of the control group in order to assess potential alterations in OA. Post-treatment samples were obtained a day after the last session of pelotherapy, to prevent the evaluation of the intervention’s immediate effects. Comparison between pre- and post-treatment values reflected the potential effect of balneotherapy on the evaluated biomarkers.

### Protocol of balneotherapy with peloids

Briefly, the spring water of the spa centre fundamentally contains bicarbonate, calcium, chloride, and sodium. The peloid is obtained from a natural stream. Afterwards, the peloid matures along with spring water for 5–8 months in a maturation tank. Peloid is composed of 39.59% of spring water and 60.41% of solid content (silt, clay, sand). A more detailed description of these elements can be found in Table 2 (Carretero et al. 2010; Pozo et al. 2013; Ortega et al. 2017).

**Table 2:** Principal physico-chemical properties and chemical composition of El Raposo waters and peloids

Water physico-chemical properties and chemical composition	
Mineralization	Medium mineralization
Flavour	Insipid
Odour	Odourless
Colour	Colourless
Spring water temperature (°C)	16.5 (hypothermal)
pH (to the water spring temperature)	7.0
Turbidity (UN)	0.0
Dry residue to 180 °C (mg/L)	591.0
Dry residue to 110 °C (mg/L)	607
Hardness (mg/L CaCO_3_)	381.9
Alkalinity (mg/L CaCO_3_)	325.0
Cl^−^ (mg/L)	71.0
F^−^ (mg/L)	0.3
HCO_3_^=^ (mg/L)	396.5
NO_3_^−^ (mg/L)	38.9
SO_4_^=^ (mg/L)	27.1
Na^+^ (mg/L)	54.9
K^+^ (mg/L)	0.7
Ca^2+^ (mg/L)	130.2
Mg^2+^ (mg/L)	13.8
Fe total (mg/L)	0.1
CO_2_ dissolved (mg/L)	10.9
Radon (Bq/L)	32
Alfa total (Bq/L)	0.18
Beta total (Bq/L)	< determination limit
Peloid physico-chemical properties	
Colour	Light brown
Water (wt.%)	39.59
Solids (wt.%)	60.41
Dry residue (850 °C)% (w/w)	53.23
Density (kg/m^3^)	1427
Hardness (g)	394
Cohesiveness	0.80
Adhesiveness (g ∙ s)	7102
Springiness (mm)	19.62
Abrasiveness (mg)	84.60
Peloid mineralogical composition	
Sand (%)	2.40
Silt (%)	53.80
Clay (%)	43.80
Phyllosilicates (wt.%)	65
Smectite (wt.%)	45
Illite (wt.%)	19
Kaolinite (wt.%)	1
Quartz (wt.%)	10
Calcite (wt.%)	20
Plagioclase (wt.%)	3
Organic matter (wt.%)	2
Peloid chemical composition	
SiO_2_ (%)	39.73
Al_2_O_3_ (%)	10.08
Fe_2_O_3_ (%)	4.49
CaO (%)	18.30
TiO_2_ (%)	0.548
MnO (%)	0.056
K_2_O (%)	1.518
MgO (%)	1.25
Na_2_O (%)	0.324
S (%)	0.10
Ba (ppm)	587.9
Ce (ppm)	74.2
Cr (ppm)	65.4
Rb (ppm)	77.8
Sr (ppm)	87.3
V (ppm)	76.5
Zn (ppm)	160.4
Zr (ppm)	130.2

The group of patients with OA underwent a daily pelotherapy session for 10 consecutive days, always in the morning by the same therapists. Briefly, as previously reported on other investigations in this spa centre (Ortega et al. 2017; Gálvez et al. 2018a, b), peloid at 40–42 °C was applied over the whole body by brush, allowing it to dry for 45–60 min in a solarium. Finally, patients received a mud bath (mixture of spring water and peloid) at 38–40 °C for 15 min, and peloid remnants were eliminated using a thermal water jet (38–40 °C) for 2 min. No adverse events were reported during this procedure.

### Sample collection

Fasting peripheral blood samples were obtained between 8.00 and 9.00 am. To isolate serum, blood samples remained at room temperature for 15–20 min after extraction to allow clotting. Blood samples for plasma isolation were anticoagulated with EDTA. Then, blood samples were centrifuged at 700 g for 10 min, and the serum and plasma samples were aliquoted and stored at − 80 °C until assay.

### Determination of systemic serotonin and dopamine concentrations

Commercial enzyme-linked immunosorbent assay (ELISA) kits were used to determine serum concentrations of serotonin (Reddot Biotech Inc., Kelowna, BC, Canada), and plasma concentrations of dopamine (Demeditec Diagnostics GmbH, Kiel, Germany). Detection limits of the serotonin and dopamine ELISA were 4.16–1000 ng/mL and 75–33,333 pg/mL, respectively. All samples were evaluated with the same kit on the same day to avoid inter-assay variations.

### Statistical analysis

The values are expressed as mean ± standard error of the mean (SEM) of the determinations performed in duplicate in each OA patient (*n* = 16) and control individuals (*n* = 8). The normal distribution of the variables was checked using the Kolmogórov-Smirnov normality test, followed by Student’s *t* test for unpaired (pre-treatment vs. control) or paired (pre-treatment vs. post-treatment) samples. Significance level was set at *p* < 0.05. Calculations were conducted with IBM® SPSS® Statistics version 28 software package.

## Results

**Fig. 2:**
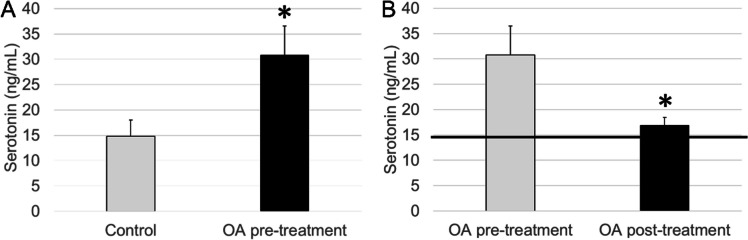
Peripheral serotonin concentrations in serum (ng/mL) in the **A** control group and OA group before balneotherapy (OA pre-treatment) and in the **B** OA group before balneotherapy (OA pre-treatment) and OA group after balneotherapy (OA post-treatment). Columns represent the mean ± SEM of independent assays performed in duplicate for each participant. *p < 0.05 with respect to **A** control values or **B** pre-treatment values. The horizontal line represents the mean serum concentration, as a reference value, determined in the age-matched control group

At baseline, OA patients showed significantly higher concentrations of circulating serotonin than healthy individuals of their same age (*p* < 0.05, Fig. 2A). After the pelotherapy cycle, peripheral serotonin concentrations were significantly reduced in OA patients (*p* < 0.05), presenting values closer to those of the control group (Fig. 2B).

**Fig. 3:**
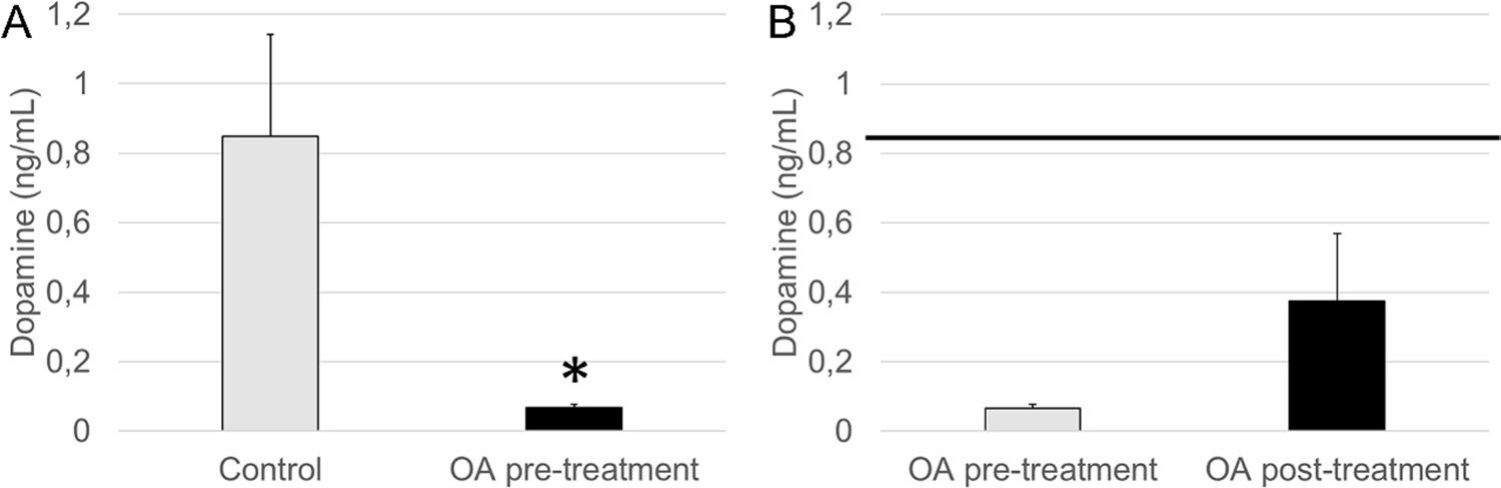
Peripheral dopamine concentrations in plasma (ng/mL) in the **A** control group and OA group before balneotherapy (OA pre-treatment) and in the **B** OA group before balneotherapy (OA pre-treatment) and OA group after balneotherapy (OA post-treatment). Columns represent the mean ± SEM of independent assays performed in duplicate for each participant. *p < 0.05 with respect to control values. The horizontal line represents the mean plasmatic concentration, as a reference value, determined in the age-matched control group

Concentrations of circulating dopamine were significantly lower in patients with OA at baseline in comparison to age-matched healthy subjects (*p* < 0.05, Fig. 3A). After the pelotherapy cycle, peripheral dopamine concentrations showed a tendency to increase in OA patients, although no significant differences were found (Fig. 3B).

## Discussion

The available literature reveals a limited number of studies that have investigated the effects of balneotherapy (or hydrotherapy, mud therapy, etc.) on serotonin or dopamine function, as shown by the systematic review performed out by our group. The studies show a high degree of variability and have low methodological rigor, posing challenges in establishing clear conclusions. In addition, no previous investigations have been found specifically examining the systemic concentrations of these neuroimmunomediators in aged patients with OA in the context of balneotherapy, providing a clear justification for conducting this preliminary pilot study.

OA is a heterogeneous disease with a broad spectrum of underlying mechanisms leading to joint destruction. OA is mainly characterized by cartilage destruction, remodelling of subchondral bone, and synovial membrane inflammation (Hunter and Bierma-Zeinstra 2019). In the complex pathogenesis of OA, inflammation and other immune processes within the joint have emerged as critical factors, with significant recognition given to both innate and adaptive immunological mechanisms (Coaccioli et al. 2022). This is due to the release of inflammatory mediators, including cytokines, which can have a significant catabolic impact on joint tissues (Scanzello and Goldring 2012; Daghestani and Kraus 2015; Hunter and Bierma-Zeinstra 2019). Systemic low-grade inflammation OA can also play a role on the onset or aggravation of the pathogenic process, and, in turn, local mediators produced within the joint can be reflected systemically, thus potentially contributing to the maintenance of this systemic inflammatory state (Berenbaum 2013; Gálvez et al. 2017). In fact, results from our laboratory have demonstrated that a dysregulation of the immune-neuroendocrine response that involves the negative feedback between the inflammatory and stress responses is present in OA, aggravating the systemic low-grade inflammatory status (Gálvez et al. 2017). High circulating concentrations of inflammatory cytokines and the eHsp72 stress protein, together with reduced cortisol levels and a reduction in neutrophil defence capacity, among other immune alterations, have been described in elderly OA patients (Gálvez et al. 2017). Novel mechanisms, such as miRNA involved in inflammation, have also revealed significant insights into OA pathogenesis (Cheleschi et al. 2018, 2022b). Moreover, pain, which represents the major symptom in OA, entails peripheral nociceptive pain mechanisms (by tissue injury or inflammation in the joint) and pain sensitisation by means of neuropathic or central pain mechanisms (Hunter and Bierma-Zeinstra 2019), including neuroinflammatory processes. Interestingly, bidirectional interactions between the immune and the nervous system are gaining recognition for their contributory role in chronic pain in OA (Coaccioli et al. 2022).

Serotonin is an essential mediator in inflammatory processes, playing a potential role in inflammatory joint disease, with pro-inflammatory effects. Furthermore, peripheral serotonin, together with other proinflammatory mediators, is involved in pain mechanisms and contributes to injury and inflammation-induced pain and hyperalgesia (Haleem 2018).

Our results show that higher serum levels of serotonin are present in OA patients compared to an age-matched group of healthy subjects, which could contribute to OA pathogenesis and progression. To the best of our knowledge, no previous studies have demonstrated elevated peripheral serotonin concentrations in OA patients in comparison to a healthy age-matched group. In line with our results, previous studies have reported that synovial fluid samples in patients with polyarthritides show high levels of serotonin, which is otherwise undetectable in healthy individuals (Alstergren et al. 1999), and that RA patients present increased levels of serotonin compared to healthy subjects, which may contribute to the excessive bone loss in this disease (Kopp and Alstergren 2002). In fact, a positive correlation was found between serum levels of serotonin and pain in these patients (Kopp and Alstergren 2002). Other studies showed that 5-hydroxyindoleacetic acid (5-HIAA, the main metabolite of serotonin), serum levels were higher in RA patients than in OA patients. However, 5-HIAA levels in synovial fluid, as well as serotonin concentrations in synovial fluid and blood, were similar in both diseases (Igari et al. 1987). Moreover, in patients with RA at advanced stages of the disease, 5-HIAA levels tended to increase both in synovial fluid and plasma (Igari and Shimamura 1979).

Regarding serotonin as a potential target for the treatment of rheumatic diseases, a study found that the 5-HT_3_ receptor antagonist tropisetron (via intra-articular injections) caused an improvement in inflammation and pain in different inflammatory rheumatic diseases, probably due to the decrease in the release of the inflammatory and pain mediator substance P, which is released after binding of serotonin to its receptor (Stratz and Müller 2000). Tropisetron has also been reported to completely block serotonin-induced overexpression of prostaglandin E2, as well as to downregulate TNF-α and IL-1β (Seidel et al. 2008). Blockade of 5-HT_2A_ receptors with ketanserin and ritanserin has also shown to limit the impact of arthritis (Pertsch et al. 1993). Therefore, blockade of serotonin action seems to have anti-inflammatory effects in inflammatory rheumatic diseases. These results are in line with the findings of the present pilot study, with a non-pharmacological intervention. The same protocol of pelotherapy assessed in the present study showed anti-inflammatory effects in OA, with an increase in cortisol concentration and a decrease in circulating pro-inflammatory cytokines concentrations, as demonstrated by studies previously conducted by our group (Ortega et al. 2017; Gálvez et al. 2018b). In the present study, we found a decrease in serotonin levels after pelotherapy, which could also be participating in the anti-inflammatory effects of the therapy, and contributing to the reduced pain levels and increased joint mobility that have been reported in these patients after mud therapy in previous investigations from our group (Ortega et al. 2017; Gálvez et al. 2018b). Thus, peripheral serotonin levels could be a good biomarker for the evaluation of the anti-inflammatory effects of balneotherapy in inflammatory rheumatic diseases such as OA.

Dopamine is also a key mediator in rheumatic pathologies since it modulates bone remodelling by affecting the differentiation of osteoclasts (inhibiting osteoclast formation) or the secretion of pro-inflammatory cytokines, with a direct effect on the systemic immune response and joint inflammation (Capellino 2020; Feng and Lu 2021). In fact, dopamine receptors in OA are differentially expressed compared to healthy control groups (Sheikhpour et al. 2020). Despite these interesting results, the potential direct role of dopamine on inflammatory rheumatic diseases has not been intensively considered until the last decade (Capellino 2020). To the best of our knowledge, no previous investigations have evaluated circulating dopamine concentrations in OA patients in comparison to a healthy age-matched group. In the present study, OA patients showed significantly lower concentrations of plasmatic dopamine than healthy individuals. The reported anti-inflammatory effects of dopamine (Lu et al. 2015) have been the basis for investigations assessing the potential role of dopamine in inflammatory rheumatic diseases treatment, such as RA and OA. Recent studies focus on the inhibition of inflammatory responses by targeting dopamine receptors to treat inflammatory diseases (Feng and Lu 2021; Schwendich et al. 2022). Capellino et al. (2014) demonstrated that exogenous dopamine strongly inhibited the production of IL-8 in RA patients, suggesting that dopaminergic agents lead to a reduction of inflammation. Accordingly, Lu et al. (2019) reported that in OA, in vitro dopamine treatment inhibited the production of inducible nitric oxide synthase, cyclooxygenase-2, matrix metalloproteinase (MMP)-1, MMP-3, and MMP-13, while increasing type II collagen and glycosaminoglycan. Furthermore, dopamine suppressed cartilage matrix degradation and reduced OA scores, presenting dopamine as a novel therapeutic agent for OA treatment (Lu et al. 2019). These findings are in line with the reduced concentrations of dopamine found in OA in the present study, which could be potentially related to the pro-inflammatory status present in these patients, which have been reported to present increased systemic concentrations of inflammatory cytokines, the stress protein eHsp72, and reduced concentrations of cortisol (Gálvez et al. 2017). Moreover, peripheral dopamine levels increased in OA patients after balneotherapy with peloids, although no statistical significance was found (but levels became closer to those of the aged-matched control group). These results could partially be in line with those obtained by Kurabayashi et al. (2001), showing an increase in dopamine levels after hydrotherapy, although it was carried out in young healthy individuals.

## Conclusions

For the first time, OA patients showed higher levels of serotonin and lower levels of dopamine than the age-matched control group, which is in agreement with the known roles of these mediators in inflammatory rheumatic diseases. After balneotherapy with peloids, serotonin concentrations were significantly reduced, and dopamine levels showed a tendency to increase, all of which could be contributing to the previously reported anti-inflammatory effects of balneotherapy. These results could have a relevant impact on OA management, because they show the therapeutic potential of endogenous stimulation/inhibition of dopamine and serotonin through non-pharmacological strategies such as balneotherapy.

Nevertheless, limitations of the present investigation are the sample size, evaluated neurohormones, and the duration of the effects after treatment. Further clinical studies with a greater sample size and follow-up evaluation will be needed to confirm a potential role of serotonin and dopamine in the pro- and anti-inflammatory effects of balneotherapy in rheumatic diseases, particularly with peloids and in elderly OA patients. In addition, it would be interesting to evaluate other neurohormones such as oxytocin, noradrenaline, etc., also considering other balneotherapy interventions.

## Data Availability

The participants of this study did not give written consent for their data to be shared publicly, so the research supporting data is not available.
